# Functional and Structural Brain Plasticity Enhanced by Motor and Cognitive Rehabilitation in Multiple Sclerosis

**DOI:** 10.1155/2015/481574

**Published:** 2015-05-06

**Authors:** Luca Prosperini, Maria Cristina Piattella, Costanza Giannì, Patrizia Pantano

**Affiliations:** ^1^Department of Neurology and Psychiatry, Sapienza University, Viale dell'Università 30, 00185 Rome, Italy; ^2^Department of Radiological Sciences, Oncology and Pathology, Sapienza University, Rome, Italy; ^3^IRCCS Neuromed, Pozzilli, Italy

## Abstract

Rehabilitation is recognized to be important in ameliorating motor and cognitive functions, reducing disease burden, and improving quality of life in patients with multiple sclerosis (MS). In this systematic review, we summarize the existing evidences that motor and cognitive rehabilitation may enhance functional and structural brain plasticity in patients with MS, as assessed by means of the most advanced neuroimaging techniques, including diffusion tensor imaging and task-related and resting-state functional magnetic resonance imaging (MRI). In most cases, the rehabilitation program was based on computer-assisted/video game exercises performed in either an outpatient or home setting. Despite their heterogeneity, all the included studies describe changes in white matter microarchitecture, in task-related activation, and/or in functional connectivity following both task-oriented and selective training. When explored, relevant correlation between improved function and MRI-detected brain changes was often found, supporting the hypothesis that training-induced brain plasticity is specifically linked to the trained domain. Small sample sizes, lack of randomization and/or an active control group, as well as missed relationship between MRI-detected changes and clinical performance, are the major drawbacks of the selected studies. Knowledge gaps in this field of research are also discussed to provide a framework for future investigations.

## 1. Introduction

Multiple sclerosis (MS) is a long-lasting disease typically affecting young adults characterized by the presence of multifocal inflammatory demyelinated plaques distributed over time and space within the central nervous system (CNS) [1]. Pathological features of MS include breakdown of the blood-brain barrier, multifocal inflammation, demyelination, oligodendrocyte loss, reactive gliosis, and various degrees of axonal damage, ranging from transient dysfunction to irreversible loss, even at early stages of the disease [2]. Although acute inflammation usually causes reversible neurological dysfunction, MS relapses may also lead to residual irreversible disability involving both motor and cognitive functions [3, 4].

In the last decade, improved diagnostic criteria and availability of effective disease-modifying drugs have led to a paradigm shift towards earlier diagnosis and treatment [5, 6]. In spite of this, either the actually available disease-modifying treatment or the other pharmacological approaches have little or no impact on permanent impairments, with motor symptoms and cognitive deficit reported, respectively, by 45–90% and 40–65% of patients throughout their disease, with a certain degree of overlap [7–9].

Consequently, the management of motor and cognitive disturbances still relies on rehabilitative strategies [10, 11], which have been reported to be effective in ameliorating these functional domains, suggesting that remediation/compensation may occur into even damaged brain structures [12]. This may imply that rehabilitation is able to enhance neuroplasticity, that is, the intrinsic property of the CNS to structurally and functionally adapt itself in response to external stimuli, environmental changes, or injuries [13]. While in healthy individuals the plasticity represents the basis of brain development, learning, and memory, in the context of MS this term encompasses molecular, synaptic, cellular events and even reorganization of the brain cortex or fibers that result in recovery of function after an acute or chronic damage [14].

The most promising advanced magnetic resonance imaging (MRI) techniques for investigating brain plasticity are the functional MRI (fMRI) and diffusion tensor imaging (DTI) [15]. The fMRI is based on the detection of changes in the blood oxygenation level-dependent (BOLD) signal, which is in turn affected by changes in neural activity in a specific brain region and the underlying physiology or pathology. Changes in BOLD signals can be investigated during the execution of a specific task (e.g., simple motor activity, sensory stimulation, and cognitive effort) [16] or at rest to explore the functional connectivity (FC), that is, the functional interaction between different brain regions [17]. The DTI is a method to assess myelin integrity* in vivo*, providing information on the integrity of the myelin-axon unit based on the directional asymmetry of water diffusion, that is, the so-called fractional anisotropy (FA) [18]. The FA value is determined by the ratio of axial diffusivity (AD) and radial diffusivity (RD), and decreased AD and increased RD are considered as markers of axonal damage and demyelination, respectively, thus representing parameters that are sensitive to underlying pathological processes of MS [19].

Using these advanced MRI techniques, it has been recently demonstrated that rapid-onset plasticity and functionally relevant chronic reorganization processes are preserved even in the most advanced stage of the disease and that these phenomena are functionally relevant to maintain motor and cognitive function [16, 20, 21].

All these findings support the hypothesis that neuroplasticity may be enhanced by rehabilitation [12]. In this view, advanced MRI may address knowledge gaps between the observed clinical improvement and the neural mechanisms underlying the improved function after rehabilitation, providing a powerful tool to investigate functional and structural brain changes related to recovery of function [22]. However, only few studies have investigated the mechanisms of rehabilitation-induced neuroplasticity so far, providing fragmented and incomplete data, in spite of the fact that rehabilitation is recognized as having a key role in the management of patients with MS [23].

Therefore, in this systematic review, we sought to summarize the existing MRI-based evidences that motor and cognitive rehabilitation may induce functional and structural plasticity into the brain of patients with MS.

## 2. Methods

*Search Strategy and Article Selection*. According to the Preferred Reporting Items for Systematic Review and Meta-Analyses (PRISMA) statement [24], two electronic databases (PubMed and Scopus) were searched for English-language articles focusing on human studies.

The search was run using the following terms: (“training” OR “rehabilitation”) AND “imaging” AND “multiple sclerosis” [All Fields]. No article type limitations or time period restrictions were applied, and the latest search was undertaken on January 20, 2015.

Attempts to identify further articles were done by searching the references of the studies. We were not familiar with any study currently in progress that could be considered for inclusion, except for one study from our group recently submitted. Published conference abstracts, articles not available in English, and those including patients also affected by neurological conditions other than multiple sclerosis were excluded.

To fit the main purpose of this systematic review, that is, summarize the existing MRI-based evidences of rehabilitation-enhanced functional and structural plasticity in MS, we also excluded studies whose training was based on short-term learning, those in which MRI was used to predict the outcome of rehabilitation, and those in which the occurrence of brain plasticity was assessed by means of techniques other than MRI (e.g., transcranial magnetic stimulation, electroencephalogram, etc.).

Abstracts of resulting articles were then examined in order to select studies that met eligibility criteria. To assess eligibility, two investigators (Surnames are provided) independently searched for articles, and agreement between them was required in order to include an article. In case of disagreement, the decision was made by the most experienced author (P. Pantano) after reading the whole article.

First author, year of publication, sample size, study design, type and duration of intervention, clinical and MRI outcome measures, and interpretation of findings were extracted from included articles and recorded on an electronic spreadsheet (M. C. Piattella).

Methodological quality of included articles was assessed using the scale developed by the Physiotherapy Evidence Database (PEDro) initiative [25]. The purpose of the PEDro scale is to determine the external validity (criterion 1), internal validity (criteria 2–9), and statistical soundness (criteria 10-11) of a study included in a systematic review. Studies scoring equal or above 9 on the PEDro scale were considered methodologically “excellent,” studies ranging from 6–8 were considered “good,” studies scoring 4-5 were of “fair” quality, studies scoring below 4 were felt to be of “poor” quality.

## 3. Results

**Figure 1:**
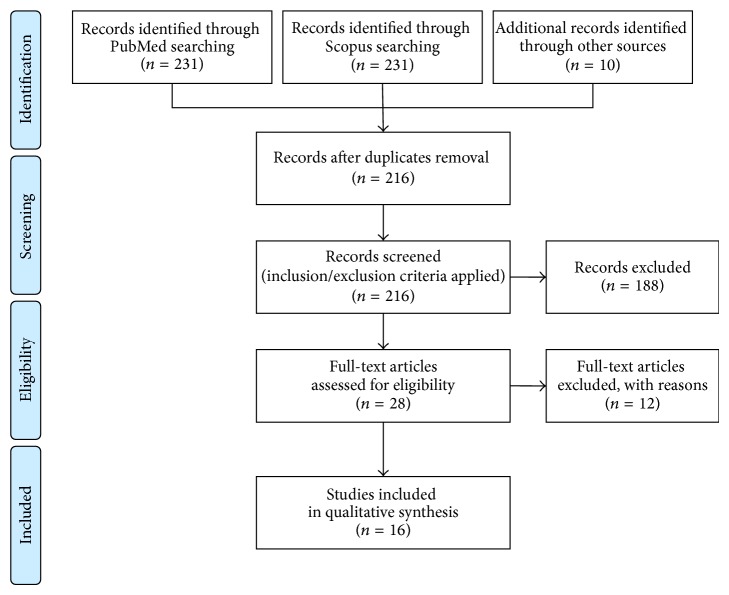
Flow diagram mapping the review according to PRISMA statement [24].

The strategy search initially yielded 216 and 231 articles in the PubMed and Scopus databases, respectively; additional 10 articles were found from other sources (references of selected papers). After applying the inclusion/exclusion criteria and checking full-text articles for eligibility, a total of 16 articles were included in the qualitative synthesis, as shown in the flow diagram in Figure 1.

Six studies investigated whether motor rehabilitation strategies enhance brain plasticity, as evaluated by either task-related fMRI (*n* = 2) [26, 27], DTI (*n* = 3) [28–30], or both techniques (*n* = 1) [31] (see Table 1).

Ten studies investigated whether cognitive rehabilitation strategies enhance brain plasticity, as evaluated by either task-related fMRI (*n* = 5) [32–35, 37] (see Table 2) or resting-state (RS)-fMRI (*n* = 4) (see Table 3) [38–41]; just one article provided findings by combining task-related fMRI and RS-fMRI with structural MRI for mapping changes in white matter (WM) and grey matter (GM) (included in Table 2) [36].

The qualitative assessment of included studies is shown in Table 4. Data from PEDro scale showed that selected article scored between 4 and 10 of 11 total points. Studies based on nonrandomized design [26–28, 31–33, 35, 40] or those without an alternative “sham” training as control group obtained lower scores [30, 36, 38, 41].

### 3.1. Brain Plasticity Enhanced by Motor Rehabilitation

The first attempt to demonstrate the occurrence of brain plasticity following motor rehabilitation was done by Rasova and colleagues [26] who selected (without randomization) 28 patients with MS. Of them, 17 received a 2-month outpatient physiotherapy program (1-hour sessions, twice per week) based on sensorimotor learning and adaptation by combining different disciplines (Vojta's reflective locomotion, Bobath concept, sensorimotor stimulation, proprioceptive neuromuscular facilitation, Burger concept, and yoga), while the remaining 11 patients did not undergo any special training. A control group of 13 healthy subjects was also enrolled to investigate whether rehabilitation might lead brain function to approach “standard” (i.e., values found in healthy group), as evaluated by task-related fMRI consisting of repetitive thumb and index flexions at a 3-second frequency, according to a visual stimulus. Each group was scanned twice at enrolment and 2 months later. Although a relevant clinical improvement was found in the active group when compared with the control group, the authors failed to demonstrate between-group differences in the amplitude of fMRI signal of four areas contributing to sensorimotor learning (primary sensorimotor cortex, supplementary motor cortex, nucleus dentatus, and putamen), as well as increased interhemispheric dependence. Moreover, there was no relationship between changes in clinical parameters and in brain activation. The authors concluded that the unpredictable course of the disease and the heterogeneous, symptom-tailored rehabilitation strategy hampered the detection of changes at group-level in fMRI activation, questioning about the appropriateness of fMRI for investigating motor plasticity.

The same group explored the impact of operator-assisted facilitation physiotherapy on microstructural properties of the corpus callosum in 11 right-handed patients with MS [28]. They were scanned in two separate occasions 1 month apart (run-in period) and then after the 2-month rehabilitation (2-hours per week). Increased callosal FA, reduced mean diffusivity (MD) and RD were found after the intervention (by approaching the values of 11 healthy controls), while no difference was observed during the run-in period. Improved scores at the Paced Auditory Serial Addition Test (PASAT) and a trend towards an improvement of the Expanded Disability Status Scale (EDSS) score are reported by the authors, but the relationship between clinical improvement and MRI changes was not reported.

More recently, a similar study was conducted to investigate the immediate and long-term effects of a 2-month motor program activation therapy (1-hour sessions, twice weekly) [31]. Patients were clinically evaluated and scanned four times to obtain DTI data of the corpus callosum and motor task-related fMRI (flexion and extension of metacarpophalangeal joints). Follow-up data were available for 12 patients who experienced a significant improvement in some clinical scales and in DTI metrics of corpus callosum (increased FA and reduced MD) immediately after and even one month after the end of the intervention, while no relevant change was found in terms of fMRI data.

Tomassini and colleagues [27] submitted 23 patients and 13 healthy controls to short-term and long-term practice of a visuomotor task. The short-term and long-term training consisted of 12-minute training, done during the first fMRI session, and 13-minute home-based sessions, once daily, for 15 days, respectively; at the end of the training, patients and healthy subjects had a second fMRI session. From a clinical standpoint, although patients performed poorer than healthy subjects in terms of overall tracking error for the visuomotor task, the MS group improved similar to healthy group after both short-term and long-term practices, regardless of MRI measures of brain damage and disability. After the long-term practice that may be considered as equivalent to a short rehabilitative intervention, a significant reduction in task-related activation of the occipital and parietal cortices was found in patients. Greater long-term clinical improvement was found to be related to smaller changes in task-related activation over time in the left superior lobule and right lateral occipital cortex, but this correlation failed to reach a statistical significance. Long-term postintervention fMRI changes observed in patients differed from those found in healthy subjects who showed reduced task-related activation only in the occipital cortex. The authors concluded that adaptive plasticity is preserved even in chronically disabled patients with MS, but this plasticity is modulated by brain systems different from those acting in healthy subjects.

To investigate the possibility that rehabilitation induces microstructural changes of WM bundles involved in voluntary motor control, Bonzano and colleagues [29] randomized (in a 1 : 1 ratio) 30 patients with MS to receive either 2-month active, task-oriented motor rehabilitation (active group) or a 2-month passive motor rehabilitation (control group) of the upper limbs (1-hour sessions, thrice per week). Before and after rehabilitation, DTI data of the corpus callosum, left and right corticospinal tracts, and left and right superior longitudinal fasciculus were obtained. After rehabilitation, the unimanual motor performance improved in both groups, while the bimanual coordination task worsened in control group and remained stable in active group. Accordingly, reduced FA and increased RD of corticospinal tracts and corpus callosum were found in the control group, but not in the active one. The authors concluded that active (voluntary), but not passive, rehabilitation preserves WM integrity of brain structures specifically involved in the trained function, thus supporting the beneficial effect of task-oriented rehabilitation.

**Figure 2:**
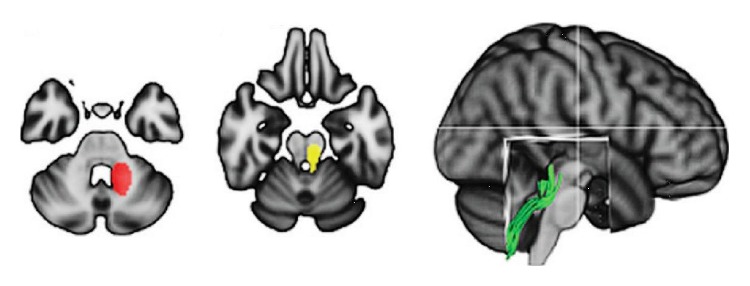
Regions of interest outlining the superior cerebellar peduncle dissected by means of streamline tractography; this white matter bundle showed significant changes indicating improved structural integrity following the 12-week home-based training using the Nintendo Wii balance board (modified from [30]).

Based on data from a randomized, two-period, crossover pilot study, showing a beneficial effect of the Nintendo Wii balance board on static balance [42], Prosperini and colleagues investigated whether DTI parameters of cerebellar connections significantly changed after intervention and whether these changes correlated with clinical improvement [30]. A total of 36 patients with MS were randomized in a 1 : 1 ratio to two counterbalanced groups: group A received 30-minute sessions, 5 days per week for 12 consecutive weeks of home-based video game training (intervention period), followed by a 12-week period without any specific intervention (observation period); group B was given the treatment in reverse order. Patients were clinically evaluated and MRI scanned at study beginning and at the end of the first and the second study periods; MRI data were available for 27 patients. Improved DTI measures of superior cerebellar peduncles were found after the training (increased FA and reduced RD), suggesting the occurrence of activity-dependent myelomodulation in partially damaged pathways (see Figure 2). These microstructural changes were also significantly related to clinical improvement of static balance, supporting the hypothesis that structural plasticity may be enhanced in brain areas specifically involved in the function trained with high-intensity, task-oriented rehabilitation. However, there was no retention of training-induced improvement in clinical and MRI measures.

### 3.2. Brain Plasticity Enhanced by Cognitive Rehabilitation

In a preliminary study, 11 patients with MS suffering from mild to severe cognitive impairment were submitted to a 3-4-week intervention with the AIXTENT software to train alertness, divided attention, selective attention, and vigilance [32]. By comparing pre- and posttraining clinical findings and task-related fMRI features, Penner and Kappos demonstrated both a clinical improvement and an increased activation of regions in the cingulate gyrus, precuneus, and frontal cortex; all these areas are known to be involved in a network functionally related to attention processing.

The effect of a mixed intervention (game-like group activities and computer-aided training) was investigated by Sastre-Garriga and colleagues [33] in an open-label, proof-of-concept trial. Fifteen patients with MS underwent an extensive neuropsychological evaluation and were scanned to obtain task-related (PASAT) fMRI before and after a 5-week run-in period. Further clinical and fMRI data were collected at the end of a 5-week cognitive training period (1-hour sessions, thrice per week). Five healthy subjects who were scanned at the same time-points served as healthy controls. After the training, patients exhibited an improved performance in backward version of digit span and increased fMRI activation in right posterior lobe (uvula and declive) and left anterior and posterior lobes of cerebellum (declive and culmen). However, clinical findings and fMRI changes did not significantly correlate, likely because the study was underpowered to detect relevant clinical changes owing the small sample size [33].

A double-blind, placebo-controlled, randomized clinical trial was designed to investigate changes in brain activation following modified Story Memory Technique (mSMT), a rehabilitative approach used for treating new learning and memory deficits [34]. A total of 16 patients were randomized either to a 5-week treatment using mSMT (active group) or to story reading and answering related questions (control group), with the same schedule (45–60-minute sessions, twice per week). Both groups were scanned to obtain task-related fMRI data during list-learning and word-recognition tasks. The proportion of patients who improved memory performance on California Verbal Learning Test short-delay free recall after the intervention was greater in the active than in the control group. Compared to controls, patients in the active group showed an increased activation in some areas of frontal, parietal, temporal, and occipital cortices and in the cerebellum. There was also a significant correlation between improved memory performance and increased activation of the right middle frontal gyrus, which is known to be associated with visual and context-dependent learning.

Some patients originally enrolled in this latter study underwent also RS-fMRI, in order to explore FC using the left and the right hippocampus (implicated in memory function) and posterior cingulated cortex (involved in the default-mode network) as seeding points [39]. Out of the two analyses performed, the less conservative one showed that after the training the active group had an increased connectivity between the left hippocampus, the insula, and pyramids of vermis, between the right hippocampus and the postcentral gyrus, and between the posterior cingulated cortex and thalamus.

Brain activation changes following a 6-week visual imagery training (2-hour sessions, once weekly) were investigated in 4 patients with MS [35]. They underwent pre- and posttraining clinical evaluations and task-related fMRI (evocation of specific personal memories). A significant improvement of autobiographic memory performance, coupled with increased activation of posterior cerebral areas specifically involved in memory retrieval (right cuneus, left precuneus, left inferior and superior occipital gyri, and left lateral temporal cortex), was found after the rehabilitation.

Cerasa and colleagues [37] performed a randomized, double-blind, controlled trial in which 26 patients with MS were allocated to receive a 6-week computer-aided training (1-hour sessions, twice per week) either with the RehaCom package, a modular system developed to treat a wide spectrum of cognitive functioning, including alertness, attention, memory, executive functions, and visual field (active group), or with a simple visuomotor coordination task (control group). Before and after the intervention, both groups were clinically examined and scanned to obtain task-related fMRI data (visual PASAT). Performance at Stroop test improved in the active group only, which also showed increased activation of brain areas subserving refreshing phonological stimuli and short-term information storage, that is, the right posterior cerebellar lobule and left superior parietal lobule.

Filippi and colleagues [36] used functional and structural MRI to investigate brain changes after a 12-week computer-assisted training with RehaCom. Twenty patients with MS were randomly allocated either to the active group (*n* = 10) or to the control group, which did not undergo any intervention (*n* = 10). Before and after the 12-week study period, both groups were assessed by a complete neuropsychological evaluation and scanned to map changes in WM and GM structures and to obtain task-related fMRI (Stroop test) and RS-fMRI data. The active group showed a clinical improvement in some tests of attention, information processing, and executive functions, an increased activation of posterior cingulated cortex and/or precuneus and dorso-lateral prefrontal cortex (bilaterally) during the task-related fMRI, and increased RS-FC of the anterior cingulated cortex (salience processing), left dorsolateral prefrontal cortex (executive function), right inferior parietal lobule, posterior cingulated cortex, and/or precuneus (default-mode network). Neither WM nor GM microarchitecture, assessed with DTI and voxel-based morphometry [43], was impacted by the rehabilitation. The authors concluded that rehabilitation of attention, information processing speed, and executive function enhance recruitment of brain networks subserving the trained functions.

In another study, data from the same population were reanalyzed using the anterior cingulated cortex as seed to explore its RS-FC [38]. At follow-up, the anterior cingulum showed an increased FC with the right inferior parietal lobule and decreased FC with the right inferior temporal gyrus in the active group only; some of these FC changes were significantly related to improved PASAT scores after the training.

Bonavita and colleagues [40] performed a nonrandomized parallel-group trial in which 18 patients were trained using the RehaCom package (active group) and 14 patients were submitted to newspaper reading and content referring for 8 consecutive weeks. Both groups underwent an extensive neuropsychological evaluation and RS-fMRI study at entry and at the end of follow-up. Several neuropsychological tests of information processing speed and verbal and visual sustained memory improved in the active, but not in the control, group after the 8-week study period. Likewise, increased RS-FC in the posterior cingulated cortex and inferior parietal cortex bilaterally (subserving the default-mode network) was found in the active group.

**Figure 3:**
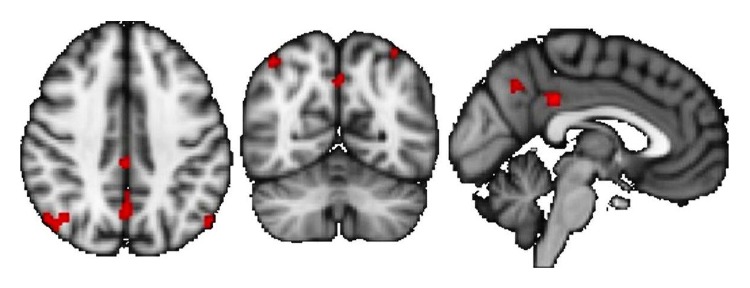
Areas of increased thalamic connectivity (posterior cingulate gyrus, precuneus, and lateral parietal cortex, bilaterally) following the 8-week home-based training using the Nintendo Dr. Kawashima Brain Training (modified from [41], courtesy of Dr. De Giglio).

Lastly, De Giglio and colleagues performed a randomized, wait-list controlled study to investigate the effectiveness of 8-week home-based playing period with the Dr. Kawashima Nintendo Brain Training, an educational video game aimed at training memory, attention, visuospatial processing, and calculations. The active group exhibited a significant improvement in sustained/divided attention and some aspects of executive functions [44]. In a post hoc analysis recently submitted for publication, 24 patients enrolled in the original trial underwent RS-fMRI before and after the cognitive training [41]. We found that this type of cognitive rehabilitative training induced an increased thalamic FC in brain areas corresponding to the posterior component of the default-mode network (cingulum, precuneus, and bilateral parietal cortex) and a decreased connectivity in the vermis and left dorsolateral prefrontal cortex (see Figure 3). Moreover, positive correlations were found between improved cognitive performance (PASAT, Symbol Digit Modalities Test, and Stroop test) and increased FC in areas belonging to the default-mode network.

## 4. Discussion

Brain plasticity represents the substrate for interventions promoting functional recovery, by means of neural restoration or compensation [12, 20, 21]. Findings from the present systematic review suggest that there is MRI-based evidence that functional or structural plasticity occurred following motor or cognitive rehabilitation in patients with MS. In addition, some studies also showed relevant relationship between improved function and MRI-detected brain changes [27, 30, 34, 36–38, 40, 41]. This latter feature supports the notion that training-induced plasticity is specifically linked to the trained function and it is not merely a general effect of any rehabilitation.

Although they differed from each other, studies on motor rehabilitation support the notion that brain plasticity is enhanced by task-dependent and target-selected training [27, 29, 30], rather than by an “holistic” approach [26]. Improved microstructural properties of corpus callosum were found following high-intensity, repetitive training of motor functions involving the lower limbs and task-oriented exercises aimed at improving upper limb functions [28, 29, 31]. Callosal fibers connect homologous cortical areas of the two hemispheres, thus subserving a wide range of motor and cognitive function, including gait and bimanual coordination [45–48]. Favourable changes in the microarchitecture of the superior cerebellar connections were reported after a video game-based training of balance [30]. Superior cerebellar peduncles mainly contain output fibers projecting from cerebellum to the neocortex, contributing to a high-level sensory weighting for postural control [49]. However, the interpretation of DTI parameters in relation to pathological changes derived is still controversial [50], and some authors argued that RD does not selectively measures demyelination due to MS but represents more complex tissue changes [51]. In addition, the reliability and sensitivity to changes of DTI measures are not well elucidated yet [19].

Studies on cognitive rehabilitation are somewhat more consistent than those on motor rehabilitation, not only in terms of trained functions but also in their results. The majority of intervention strategies consisted of computer-assisted training of attention, short-term memory, and executive functions [32, 33, 36–38, 40, 41]. Despite some differences regarding the neuropsychological scales and clinical outcome measures adopted, task-related fMRI and RS-fMRI findings are quite consistent, pointing out the role of some specific brain regions such as the cingulated cortex [32, 34, 36, 38, 40, 41], precuneus [32, 34–36, 41], and cerebellum [33, 34, 37, 39]. The cingulated cortex is known to cover emotion formation and processing, learning, and memory, thus linking behavioral outcomes to motivational learning [52–54]. The precuneus is involved in episodic memory and visuospatial imagery and it has been suggested to be a specific target for visual mirror therapy and virtual reality-based rehabilitation [55, 56]. Being connected with many association networks [57], the cerebellum has been now recognized to be not only involved in motor planning and learning, but also in different cognitive domains, including attention, memory, and learning, executive control, language, and visuospatial function [58, 59].

Despite some encouraging findings reported above, the studies included in the present systematic review suffer from several drawbacks, mainly concerning the small sample size and the absence of a nonactive control group, blindness, and/or randomization in the study design. Moreover, the selected articles are not comparable because clinical outcome measures, MRI biomarkers, and intervention are not standardized.

Only few articles report data about the occurrence of acute relapses and disability progression [28, 30, 37, 38, 41] or information on disease-modifying and symptomatic treatments taken by patients while on study [28, 29]. However, this should have not biased findings/interpretation of the included studies for several reasons: (i) no patients relapsed or experienced disability progression (when reported) [28, 30, 37, 38, 41]; (ii) it is very unlikely that relapses and disability progression might have occurred, given the short duration of the studies (from a minimum of 15 days to a maximum of 24 weeks) in the remaining studies [26, 27, 29, 31–36, 39, 40]; (iii) as per inclusion criteria only patients in a stable phase of the disease were enrolled; (iv) the randomization procedure (when applied) should have prevented any imbalance in known and unknown baseline characteristics between treatment and control groups [29, 30, 34, 36–39, 41].

The lack of statistical inferences aimed at exploring correlations between imaging results and clinical outcomes represents another major limit of some studies [26, 28, 29, 31, 32, 34, 35, 40] since there is recommendation that MRI changes following the rehabilitative interventions should be quantified and compared with clinically relevant, sensitive, and reproducible outcomes [20, 60].

Postintervention study phases were planned only rarely, but they may provide important information about the retention of rehabilitation-induced clinical and MRI improvements, especially for defining the most appropriate duration and timing of rehabilitation. Therefore, efforts for future research should be focused on establishing (i) the most appropriate strategies for effective rehabilitation; (ii) standardised, valid, and reliable endpoints to assess the efficacy of rehabilitation, taking into account the concept of ecological validity and patient-centered outcomes; and (iii) clinical and MRI measures that most effectively detect the occurrence of beneficial brain plasticity after specific training.

Another new intriguing field of research that is worth developing encompasses the possibility of combining rehabilitation with pharmacologic treatments or neuromodulation, to obtain a synergistic or even a more than additive effect on brain plasticity, as demonstrated in other pathological conditions [61–63].

In conclusion, the current knowledge about the rehabilitation-induced brain plasticity in MS is still fragmented and incomplete. The ultimate goal should be to demonstrate, at an evidence-based level, that effective rehabilitation favourably affects the brain structures, improves the trained function, and promotes the patient's quality of life.

## Figures and Tables

**Table 1:** Summary of studies investigating the effect of motor rehabilitation or training on brain plasticity, assessed by nonconventional functional or structural MRI techniques.

Authors (year)	Sample size	Main clinical characteristics	Study design	Intervention(s) and setting [setting and schedule]	Clinical outcome(s)	MRI study outcome(s)
Rasova et al. (2005) [26]	28 (13)^∗^	N/R	Non-randomized parallel group trial	**Active group:** outpatient eclectic sensori-motor learning and adaptation [1-hour sessions, 2 times per week, for 2 months] **Control group:** no special exercise (MS)	The 9-HPT, 25-FWT, PASAT, postural reactions, MS QoL-54, and BDI improved in active group	No changes detectable by task-related fMRI

Ibrahim et al. (2011) [28]	11 (11)^∗^	Mean age: ~43 years Mean MS duration: ~6 years Median EDSS: 3.5 Course: 11 RR	Non-randomized pre-post comparison study	Operator-assisted facilitation physiotherapy [2-hour sessions, once a week, for 2 months]	PASAT improved after the intervention	Significant increase of FA and decrease in MD and RD were observed after the intervention

Tomassini et al. (2012) [27]	23 (12)^∗^	Mean age: ~45 years Mean MS duration: ~12 years Median EDSS: 4.0 Course: N/R	Non-randomized pre-post comparison study	Home-based visuo-motor task training [12-minute sessions, once a day, for 15 days]	Overall tracking error during the visu-motor task execution decreased afte the training	After the training, a significant reduction in fMRI activation was observed in the occipital and parietal cortices

Bonzano et al. (2014) [29]	30	Mean age: ~43 years Mean MS duration: ~18 years Median EDSS: 4.0 Course: 22 RR, 18 SP	Randomized controlled trial	**Active group:** outpatient active motor rehabilitation of upper limbs [1-hour sessions, 3 times per week, for about 2 months] **Control group:** outpatient passive motor rehabilitation of upper limbs [1-hour sessions, 3 times per week, for about 2 months]	Both groups improved on unimanual motor performance, but bimanual coordination worsened in control group	Reduced FA and increased RD of corticospinal tracts and corpus callosum were found in control group, as detected by DT-MRI measures

Prosperini et al. (2014) [30]	27	Mean age: ~36 years Mean MS duration: ~10 years Median EDSS: 3.0 Course: 26 RR, 1 SP	Randomized two-period cross-over trial	**Active group:** home-based video game balance board [30-minute sessions, 5 times per week for 12 weeks] **Control group:** no intervention	Static balance detected at static posturography improved in active group	Increased FA and reduced RD of superior cerebellar peduncles were found in active group, as detected by DT-MRI; DTI changes were significantly related to improved static balance

Rasova et al. (2015) [31]	12	Mean age: ~40 years Mean MS duration: ~7 years Median EDSS: 3.5 Course: 11 RR, 1 PP	Non-randomized uncontrolled comparison trial	Motor programme activating therapy [1-hour sessions, 2 times per week, for about 2 months]	The MAS, 25-FWT, 9-HPT, and cerebellar functions improved immediately after and one month apart from the end of rehabilitation	Increased FA and reduced MD of corpus callosum immediately after and one month apart from the end of rehabilitation; no changes were detected with task-related fMRI

9-HPT: 9-hole peg test; 25-FWT: 25-foot walking test; BDI: Beck Depression Inventory; DTI: diffusion tensor imaging; EDSS: Expanded Disability Status Scale; fMRI: functional magnetic resonance imaging; FA: fractional anisotropy; MAS: Modified Ashworth Scale; MD: mean diffusivity; MS QoL-54: 54-item Multiple Sclerosis Quality of Life; N/R: not reported; PASAT: Paced Auditory Serial Addition Test; PP: primary progressive; RR: relapsing-remitting; SP: secondary progressive.

^∗^The number within parentheses refers to the sample size of healthy subjects.

**Table 2:** Summary of studies investigating the effect of cognitive rehabilitation or training on brain plasticity, assessed by task-related fMRI.

Authors (year)	Sample size	Main clinical characteristics	Study design	Intervention(s) and setting [intervention schedule]	Clinical outcome(s)	MRI outcome(s)
Penner and Kappos (2006) [32]	11	N/R	Nonrandomized pre-/postcomparison study	Computer-assisted attention training with the AIXTENT package [3-4 weeks]	Not reported	Increased activation of regions in the cingulate gyrus, precuneus, and frontal cortex was found after the training

Sastre-Garriga et al. (2011) [33]	15 (5)^∗^	Mean age: ~51 years Mean MS duration ~14 years Median EDSS: 6.0 Course: SP 10, RR 3, and PP 2	Nonrandomized pre-/postcomparison study	Mixed intervention: computer-assisted cognitive rehabilitation and game-like group activities [1-hour sessions, 3 times per week, for 5 weeks]	Improvement in digit span after the intervention	Increased activation of right posterior cerebellar lobe and left anterior and posterior cerebellar lobe after the intervention

Chiaravalloti et al. (2012) [34]	16	Mean age: ~48 years Mean MS duration ~15 years Median EDSS: N/R Course: 13 RR, 2 PP, and 1 SP	Double-blind randomized controlled group	**Active group: **modified story memory technique [45–60-minute sessions, 2 times per week, for 5 weeks] **Control group:** story reading and answering questions [45–60-minute sessions, 2 times per week, for 5 weeks]	CVLT short-delay free recall improved in active group	Increased activation of some areas of frontal, parietal, temporal, and occipital lobes and cerebellum in active group

Ernst et al. (2012) [35]	8	Mean age: ~38 years Mean MS duration ~14 years Median EDSS: 2.0 Course: 8 RR	Nonrandomized controlled trial	**Active group:** Motor visual imagery training [2-hour sessions, 1 time per week, for 6 weeks] **Control group:** no intervention	Autobiographic memory improved in active group	Increased activation of some areas located in posterior regions (right cuneus, left precuneus, left inferior and superior occipital gyri, and left lateral temporal cortex)

Filippi et al. (2012) [36]	20	Mean age: ~46 years Mean MS duration ~14 years Median EDSS: 2.0 Course: 20 RR	Randomized controlled trial	**Active group:** outpatient cognitive training using the RehaCom package [1-hour sessions, 3 times per week, for 12 weeks] **Control group:** no intervention	The PASAT, WCST, and oral word association test improved in active group	(i) Increased activation of posterior cingulated cortex and/or prefrontal cortex (bilaterally) in active group; (ii) increased activation at rest of anterior cingulated cortex (salience processing), left dorsolateral prefrontal cortex (executive function), right inferior parietal lobule and posterior cingulated cortex, and/or precuneus (default-mode networks I and II); (iii) no significant WM and GM changes

Cerasa et al. (2013) [37]	23	Mean age: ~32 years Mean MS duration ~9 years Median EDSS: 2.5 Course: 23 RR	Double-blind randomized controlled trial	**Active group:** outpatient cognitive training using the RehaCom package [1-hour sessions, 2 times per week, for 6 weeks] **Control group:** home-based training with a simple visuomotor coordination task (in-house software) [1-hour sessions, 2 times per week, for 6 weeks]	The Stroop test improved in active group	Increased activation of the right posterior cerebellar lobule and left superior parietal lobule in active group

CVLT: California Verbal Learning Test; GM: grey matter; N/R: not reported; PASAT: Paced Auditory Serial Addition Test; PP: primary progressive; RR: relapsing-remitting; SP: secondary progressive; WCST: Wisconsin Card Sort Test; WM: white matter.

^∗^The number within parentheses refers to the sample size of healthy subjects.

**Table 3:** Summary of studies investigating the effect of cognitive rehabilitation or training on brain plasticity, assessed by RS-fMRI.

Authors (year)	Sample size	Main clinical characteristics	Study design	Intervention(s) and setting [setting and schedule]	Clinical outcome(s)	MRI study outcome(s)
Parisi et al. (2014) [38]	20	Mean age: ~46 years Mean MS duration ~14 years Median EDSS: 2.0 Course: 20 RR	Randomized controlled trial	**Active group:** outpatient cognitive training using the RehaCom package [1-hour sessions, 3 times per week, for 12 weeks] **Control group:** no intervention	The PASAT, WCST, and oral word association test improved in active group	Increased connectivity of the anterior cingulum with the right inferior parietal lobule and decreased connectivity of the anterior cingulum with the right inferior temporal gyrus were found in active group

Leavitt et al. (2012) [39]	14	Mean age: ~49 years Mean MS duration ~14 years Median EDSS: N/R Course: 9 RR, 3 SP, and 2 PP	Double-blind randomized controlled group	**Active group: **modified story memory technique [45–60-minute sessions, 2 times per week, for 5 weeks] **Control group:** story reading and answering relative questions [45–60-minute sessions, 2 times per week, for 5 weeks]	Just a nonsignificant improvement of CVLT short-delay free recall was found in active group	Increased connectivity (default-mode network) of insula and pyramids of vermis (left hippocampus seeded), postcentral gyrus (right hippocampus seeded), and thalamus (posterior cingulated cortex seeded)

Bonavita et al. (2015) [40]	32	Mean age: ~47 years Mean MS duration ~21 years Median EDSS: 4.5 Course: 32 RR	Nonrandomized parallel group trial	**Active group:** outpatient cognitive training using the RehaCom package [50-minute sessions, 2 times per week for 8 weeks] **Control group:** newspaper reading and content referring [30-minute sessions, 2 times per week, for 8 weeks]	The SDMT, PASAT, SRT-D, and SPART-D improved in active group	Increased functional connectivity (default-mode network) of the posterior cingulated cortex and inferior parietal cortex (bilaterally) was found in active group

De Giglio et al. (2015) [41]	24	Mean age: ~42 years Mean MS duration ~13 years Median EDSS: 2.0 Course: 24 RR	Randomized wait-list controlled study	**Active group:** home-based training with an educational video game [30-minute sessions, 5 days per week, for 8 weeks] **Control Group:** wait-list	Improved PASAT and Stroop was found in active group	Increased thalamocortical connectivity was found in brain areas corresponding to the posterior component of the default-mode network (thalami seeded)

CVLT: California Verbal Learning Test; PASAT: Paced Auditory Serial Addition Test; SDMT: Symbol Digit Modalities Test; SPART-D: Spatial Recall Test-delayed recall; SRT-D: Selective Reminding Test-delayed recall; WCST: Wisconsin Card Sort Test.

**Table 4:** Included articles rating according to the PEDro scale [25].

		#1	#2	#3	#4	#5	#6	#7	#8	#9	#10	#11	Score
Articles on motor rehabilitation	Rasova et al. (2005) [26]	✓	*✗*	*✗*	✓	*✗*	*✗*	*✗*	✓	✓	✓	✓	6/11
	Ibrahim et al. (2011) [28]	✓	*✗*	*✗*	*✗*	*✗*	*✗*	✓	✓	✓	✓	✓	6/11
	Tomassini et al. (2012) [20]	✓	*✗*	*✗*	*✗*	*✗*	*✗*	*✗*	✓	✓	N/A	✓	5/11
	Bonzano et al. (2014) [29]	✓	✓	✓	✓	*✗*	*✗*	*✗*	✓	✓	✓	✓	8/11
	Prosperini et al. (2014) [30]	✓	✓	✓	✓	*✗*	*✗*	✓	*✗*	✓	✓	✓	8/11
	Rasova et al. (2015) [31]	✓	*✗*	*✗*	*✗*	*✗*	*✗*	*✗*	✓	✓	*✗*	✓	4/11

Articles on cognitive rehabilitation	Penner and Kappos (2006) [32]	✓	*✗*	*✗*	*✗*	*✗*	*✗*	*✗*	✓	✓	N/A	✓	4/11
	Sastre-Garriga et al. (2011) [33]	✓	*✗*	*✗*	*✗*	*✗*	*✗*	✓	✓	✓	N/A	✓	5/11
	Chiaravalloti et al. (2012) [34]	✓	✓	✓	✓	✓	*✗*	✓	✓	✓	✓	✓	10/11
	Ernst et al. (2012) [35]	✓	*✗*	*✗*	*✗*	*✗*	*✗*	*✗*	✓	✓	✓	✓	5/11
	Filippi et al. (2012) [36]	✓	✓	✓	✓	*✗*	*✗*	✓	✓	✓	✓	✓	9/11
	Leavitt et al. (2012) [39]	✓	✓	✓	✓	✓	*✗*	✓	✓	✓	✓	✓	10/11
	Cerasa et al. (2013) [37]	✓	✓	✓	✓	✓	*✗*	✓	✓	✓	✓	✓	10/11
	Parisi et al. (2014) [38]	✓	✓	✓	✓	*✗*	*✗*	✓	✓	✓	✓	✓	9/11
	Bonavita et al. (2015) [40]	✓	*✗*	*✗*	✓	*✗*	*✗*	✓	✓	✓	✓	✓	7/11
	De Giglio et al. (2015) [41]	✓	✓	✓	✓	*✗*	*✗*	✓	*✗*	✓	✓	✓	7/11

N/A: data not available.

Criterion 1: specified eligibility criteria.

Criterion 2: randomized allocation.

Criterion 3: concealed allocation.

Criterion 4: similarity between groups at baseline.

Criterion 5: blinding of subjects.

Criterion 6: blinding of therapists.

Criterion 7: blinding of assessors.

Criterion 8: outcome measures obtained from at least 85% of initially allocated subjects.

Criterion 9: all received treatment, or key outcome, was analyzed by “intention-to-treat.”

Criterion 10: between-group statistical comparison.

Criterion 11: both point and variability measures provided.
